# PTEN, a Barrier for Proliferation and Metastasis of Gastric Cancer Cells: From Molecular Pathways to Targeting and Regulation

**DOI:** 10.3390/biomedicines8080264

**Published:** 2020-08-03

**Authors:** Milad Ashrafizadeh, Masoud Najafi, Hui Li Ang, Ebrahim Rahmani Moghadam, Mahmood Khaksary Mahabady, Amirhossein Zabolian, Leila Jafaripour, Atefe Kazemzade Bejandi, Kiavash Hushmandi, Hossein Saleki, Ali Zarrabi, Alan Prem Kumar

**Affiliations:** 1 Department of Basic Science, Faculty of Veterinary Medicine, University of Tabriz, Tabriz 5166616471, Iran; dvm.milad73@yahoo.com; 2 Radiology and Nuclear Medicine Department, School of Paramedical Sciences, Kermanshah University of Medical Sciences, Kermanshah 6715847141, Iran; najafi_ma@yahoo.com; 3 Cancer Science Institute of Singapore and Department of Pharmacology, Yong Loo Lin School of Medicine, National University of Singapore, Singapore 119077, Singapore; e0336095@u.nus.edu; 4 Department of Anatomical Sciences, School of Medicine, Student Research Committee, Shiraz University of Medical Sciences, Shiraz 7134814336, Iran; e_rahmani@sums.ac.ir; 5 Kazerun Health Technology Incubator, Shiraz University of Medical Sciences, Shiraz 6461665145, Iran; 6 Anatomical Sciences Research Center, Institute for Basic Sciences, Kashan University of Medical Sciences, Kashan 8715988141, Iran; Khaksary-m@kaums.ac.ir; 7 Young Researchers and Elite Club, Tehran Medical Sciences, Islamic Azad University, Tehran 1916893813, Iran; Fzr2000_0007@yahoo.com (A.Z.); Atefe.kazemzade@yahoo.com (A.K.B.); hosseinsaleki2015@gmail.com (H.S.); 8 Department of Anatomy, School of Medicine, Dezful University of Medical Sciences, Dezful 3419759811, Iran; elahejafari62@gmail.com; 9 Department of Food Hygiene and Quality Control, Division of Epidemiology & Zoonoses, Faculty of Veterinary Medicine, University of Tehran, Tehran 1417414418, Iran; houshmandi.kia7@ut.ac.ir; 10 Sabanci University Nanotechnology Research and Application Center (SUNUM), Tuzla 34956, Istanbul, Turkey; 11 Center of Excellence for Functional Surfaces and Interfaces (EFSUN), Faculty of Engineering and Natural Sciences, Sabanci University, Tuzla 34956, Istanbul, Turkey

**Keywords:** gastric cancer, phosphatase and tensin homolog (PTEN), PI3K/Akt, metastasis, growth

## Abstract

Cancer is one of the life-threatening disorders that, in spite of excellent advances in medicine and technology, there is no effective cure for. Surgery, chemotherapy, and radiotherapy are extensively applied in cancer therapy, but their efficacy in eradication of cancer cells, suppressing metastasis, and improving overall survival of patients is low. This is due to uncontrolled proliferation of cancer cells and their high migratory ability. Finding molecular pathways involved in malignant behavior of cancer cells can pave the road to effective cancer therapy. In the present review, we focus on phosphatase and tensin homolog (PTEN) signaling as a tumor-suppressor molecular pathway in gastric cancer (GC). PTEN inhibits the PI3K/Akt pathway from interfering with the migration and growth of GC cells. Its activation leads to better survival of patients with GC. Different upstream mediators of PTEN in GC have been identified that can regulate PTEN in suppressing growth and invasion of GC cells, such as microRNAs, long non-coding RNAs, and circular RNAs. It seems that antitumor agents enhance the expression of PTEN in overcoming GC. This review focuses on aforementioned topics to provide a new insight into involvement of PTEN and its downstream and upstream mediators in GC. This will direct further studies for evaluation of novel signaling networks and their targeting for suppressing GC progression.

## 1. Introduction

Cancer is still a leading cause of death worldwide, and its epidemiology is changing based on alterations in risk factors, its classification, advances in therapy and detection, and finally, demographic changes, such as aging, immigration, and population growth [1]. Regardless of the incidence rate of cancer, treatment of cancer requires an integrated plan to reveal all dimensions of a certain cancer to know how each cancer cell is able to ensure its survival. In spite of progresses in different fields such as cancer immunotherapy, radiotherapy, and chemotherapy that have been beneficial in improving life quality and overall survival of patients with cancer, overcoming cancer needs more advancement. Two factors, namely uncontrolled growth and metastasis of cancer cells in malignant tumors, should be considered to provide effective treatment for patients [2,3,4,5,6]. In malignant tumor, strategies such as chemotherapy, radiotherapy, immunotherapy, and surgery are just able to improve overall survival of patients and cannot completely eradicate cancer cells. Thus, studies have been directed toward molecular targeting of cancer cells [7,8,9,10,11]. Consequently, identification of molecular profile of each cancer can provide a milestone progress in achieving an effective treatment [12,13,14]. Recently, we demonstrated how phosphatase and tensin homolog (PTEN) signaling can target bladder cancer cells and how they can be regulated by other molecular pathways [15]. Since PTEN is a well-known target in cancers, and a high number of studies have focused on revealing its role in different cancers, we decided to provide another review with a focus on gastric cancer (GC). In the present review, we focus on PTEN as an onco-suppressor signaling that undergoes downregulation in gastric cancer (GC) cells. Our aim is to reveal signaling networks involved in regulation of PTEN in GC and how PTEN activation can significantly improve survival of patients with GC. This review can provide a new insight toward targeting PTEN in future studies, using pharmacological or genetic interventions, to obtain a solution for GC treatment.

## 2. PTEN Signaling: An Overview

Since expression of PTEN undergoes downregulation in cancer cells, it has attracted much attention in the field of cancer therapy [16,17,18,19,20,21,22,23]. PTEN was first discovered in 1997 when mutations were investigated at 10q23 locus in chromosome 10, and it was found that PTEN is an onco-suppressor gene [24]. Structurally, PTEN gene has nine exons, and it encodes a 403-aminoacid protein that has both protein and lipid phosphatase roles [25,26]. PTEN has five functional domains [27,28]: (1) an N-terminal phosphatidylinositol 4-5-diphosphate (PIP2)-binding domain, (2) a phosphatase domain, (3) a membrane-targeting C2 domain, (4) a C-terminal tail, and (5) a PDZ binding motif. Reduction in expression of PTEN can result in carcinogenesis, and uncontrolled proliferation and metastasis of cancer cells [29,30]. Increased malignancy of cancer cells after PTEN deletion is due to activation of the PI3K/Akt signaling pathway [31,32,33]. In fact, PTEN is an inhibitory of the aforementioned signaling network, and downregulation of PTEN leads to activation of the PI3K/Akt signaling pathway and increased proliferation of cancer cells [34,35,36,37,38,39]. Thus, before discussing the role of PTEN signaling in cancer malignancy, it would be beneficial to describe PTEN at the molecular level.

**Figure 1:**
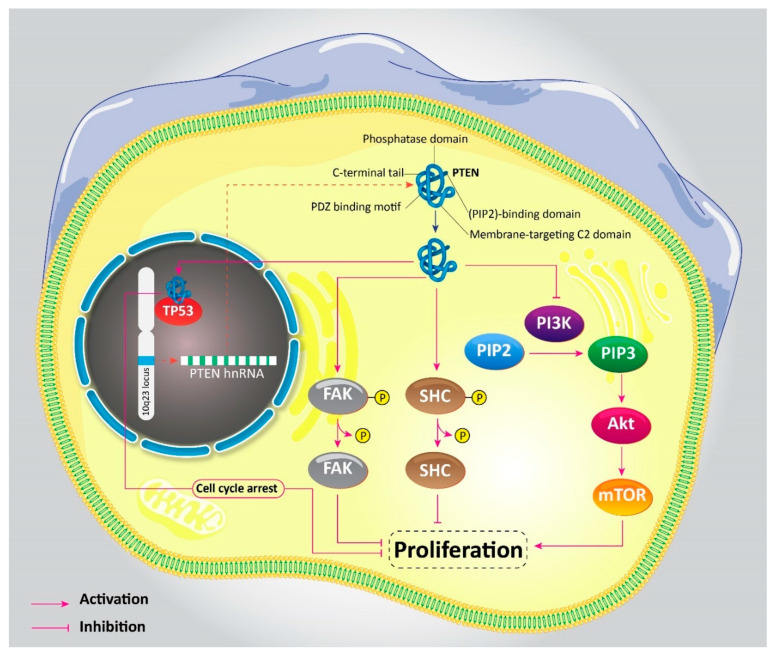
Phosphatase and tensin homolog (PTEN) signaling pathway and its interactions.

PTEN is involved in the stimulation of cell cycle arrest at G1 phase via inhibiting formation of PIP3 and reducing its level via conversing PIP3 to PIP2 [40]. PI3K induces PIP3 formation from PIP2 to activate the Akt signaling pathway. By suppressing PIP3 formation, PTEN exerts an inhibitory effect on the PI3K/Akt axis. The next step is the activation of mTOR signaling, which promotes both the migration and proliferation of cancer cells [41,42]. Thus, disrupting PI3K/Akt/mTOR signaling by PTEN leads to a dramatic decrease in survival and metastasis of cancer cells [43,44,45]. This is the pathway for lipid phosphatase activity of PTEN. In exerting protein phosphatase activity, PTEN dephosphorylates FAK and SHC proteins to negatively affect cell survival, proliferation, and invasion of cancer cells. Moreover, PTEN promotes chromosome stability by binding into TP53 in the nucleus. This interaction of PTEN with TP53 also mediates cell cycle arrest [46]. Figure 1 provides a schematic representation of PTEN signaling.

## 3. PTEN Pathway in Oncology

Inactivation of PTEN can occur at different levels, such as genetic mutation, transcriptional, and post-transcriptional levels [47]. Moreover, epigenetic mechanisms can inhibit PTEN expression via promoter hyper-methylation and histone acetylation. It is worth mentioning that activity and subcellular localization of PTEN can be affected by ubiquitination, phosphorylation, acetylation, oxidation, and sumoylation, leading to tumorigenesis [48,49,50,51,52]. Loss of PTEN expression is correlated with advanced tumor stage and resistance of cancer cells to chemotherapy [53]. In different cancers, such as breast, prostate, and endometrium cancers, PTEN can be considered as a prognostic and predictive biomarker for the response of cancer cells into chemotherapy [54]. The PTEN gene has a difference compared to classical onco-suppressor genes. Although classical onco-suppressor genes require complete silencing to stimulate carcinogenesis, a slight reduction in PTEN expression can lead to tumorigenesis [55]. The effect of partial loss of PTEN and its association with tumorigenesis have been investigated in hypomorphic mouse models [25,56,57]. PTEN activation can mediate resistance to ionizing radiation therapy. This is due to the significant role of PTEN in promoting chromatin stability and enhancing DNA repair, not the oncogene role of PTEN. After induction of DNA damage, phosphorylation of PTEN occurs at tyrosine240, and, in turn, PTEN attaches to chromatin. Then, it recruits RAD51 to enhance DNA repair [54]. Another study also demonstrates that exposing into radiation is correlated with the loss of heterozygosity of TP53 and PTEN and predisposing into tumorigenesis [58]. More studies are needed to clarify expression of PTEN during radiation therapy.

Thus, activation of PTEN can be considered as a promising strategy in cancer therapy. It seems that the downregulation of transient receptor potential vanilloid type 4 (TRPV4) provides condition for stimulation of PTEN and suppressing progression of colon cancer cells [59]. The loss of PTEN inhibits its interaction with NKX3.1 in the nucleus. As a consequence, NKX3.1 undergoes degradation, resulting in increased malignancy of cancer cells [60]. Different factors function as upstream mediators of PTEN in cancer cells. It seems that, in bladder cancer, PTEN is inhibited by nuclear factor-kappaB (NF-ĸB) signaling cascade, which can promote tumorigenesis [61,62,63,64]. USP13 suppresses NF-ĸB to promote PTEN expression, resulting in antitumor activity [65]. Long non-coding RNA (lncRNA) TUSC8 stimulates PTEN expression via miR-641 downregulation, to inhibit the progression of cervical cancer cells [66]. Thus, PTEN is a potential onco-suppressor factor, and its downregulation can facilitate progression of cancer cells [67].

## 4. PTEN in Clinical Studies

Clinical studies have also shown the onco-suppressor role of PTEN, and its downregulation in cancer cells. In an experiment conducted on men with prostate cancer, it was found that PTEN loss is associated with metastasis and invasion of cancer cells, and undesirable prognosis [68]. In another experiment in China, on breast cancer patients, occurrence of PTEN mutation is 4.8% [69]. PTEN can be used as a biomarker for response of patients with cancer into chemotherapy. Moreover, PTEN loss is related to pathologic complete response (pCR) [70]. A study conducted on colorectal patients (198 patients from 2006 to 2008 that enrolled in Taipei Veterans General Hospital) extensively examined different aspects of the PTEN gene in these patients. Mutation in the PTEN gene and the inhibition of its protein expression, inducing promoter hypermethylation and reduced DNA copy number, occur in patients with colorectal cancer. This study revealed that loss of PTEN is more common in the advanced stage (stage IV, 56.9% loss), compared to the initial stage (stage I, 20% loss). However, it was shown that PTEN deactivation has no effect on prognosis [71]. Although this experiment showed PTEN is not correlated with prognosis of colorectal patients, another study demonstrates that PTEN can be considered as a reliable biomarker for prognosis of patients with head and neck cancer, so that PTEN loss shows poor survival and prognosis [72]. These studies demonstrate there is no certain role for PTEN in clinical studies. This story is more complicated by study of Stern and colleagues. They showed that PTEN loss occurs in patients with breast cancer, but it is not associated with chemoresistance [73]. Thus, more clinical studies are required to examine the special role of PTEN in cancer patients. It seems that PTEN has a different role in each cancer type, and this role can be more specified by considering the age, sex, and race of patients with cancer [74,75].

## 5. PTEN and Gastric Cancer

Epigenetic Regulation of PTEN.

### 5.1. MicroRNAs Target PTEN

Briefly, miRs belong to non-coding RNAs (ncRNAs) from the subcategory of regulatory RNAs [76,77,78]. Although miRs comprise a large section of genome and are not translated into proteins, they exert regulatory effects on important cellular events, such as proliferation, migration, and so on [79,80,81,82,83,84,85,86]. In terms of regulation of the PTEN signaling pathway, miRs have demonstrated great potential. Increasing evidence demonstrates that miRs are able to dually downregulate and upregulate PTEN in cancer cells [69,87,88,89,90,91]. In the following sections, we focus on the relationship between miRs and PTEN signaling in GC cells.

#### 5.1.1. MicroRNAs and PTEN Induction

Metastasis of GC cells into neighboring and distant tissues is an increasing challenge. Understanding underlying molecular pathways involved in this malignancy can pave the road to effective GC therapy. Thanks to newly conducted researches in this field, it has been reported that epithelial-to-mesenchymal transition (EMT) and angiogenesis are two vital factors that promote the migration of GC cells. Transformation of epithelial cells into mesenchymal ones is performed during the EMT mechanism, with a reduction in E-cadherin levels (epithelial marker) and an increase in mesenchymal markers (N-cadherin and vimentin) [92,93]. On the other hand, angiogenesis not only provides energy and oxygen supplies for cancer cells, but also directs cancer cells into blood circulation and their migration into distant locations [94,95,96]. Thus, these two mechanisms are critical for cancer cells and harmful for patients with cancer. The PI3K/Akt signaling pathway can facilitate EMT and angiogenesis in cancer cells [97,98], so targeting PI3K/Akt and its upstream mediators is of importance in cancer therapy. In GC cells, the aforementioned signaling networks are controlled by miRs. Overexpression of miR-616-3p stimulates the PI3K/Akt signaling pathway via PTEN downregulation. This accelerates the stimulation of angiogenesis via upregulation of VEGFA/VEGFR2 axis. Moreover, miR-616-3p inhibits PTEN to enhance expressions of snail, slug, and vimentin, along with a substantial decrease in E-cadherin expression [99]. In addition to migration and invasion, miRs can participate in the resistance of GC cells into apoptosis by targeting PTEN signaling. In order to improve overall survival of patients with cancer and simultaneously suppress their malignancy, induction of apoptosis is an ideal candidate. MiR-575, as an oncogene factor, reduces the expression of PTEN, to diminish apoptosis in GC cells [92]. Although this study has not examined the expression of PI3K/Akt, it appears that resistance of GC cells into apoptosis in previous studies was due to activation of the PI3K/Akt signaling pathway.

As a miR with antitumor activity, miR-136 can induce apoptosis via downregulation of Bcl-2 [100]. In addition to proliferation, miR-136 interferes with migration of GC cells via the inhibition of HOXC10 [101]. Although these studies advocate for an onco-suppressor role of miR-136 in GC, a recent study has confirmed that miR-136 functions as an oncogene factor via targeting PTEN in GC cells. It seems that miR-136 downregulates the expression of PTEN, to elevate the viability and migration of GC cells. It is worth mentioning that miR-136 has no effect on Akt, and it just affects phosphorylated Akt in GC cells [102]. Therefore, targeting such miRs can lead to suppressing GC progression and development. An experiment has performed such a strategy in GC therapy. Silencing miR-21 is associated with stimulation of apoptosis and cell cycle arrest in GC cells. This is due to the upregulation of PTEN at the protein level and subsequent inhibition of Akt phosphorylation at Thr308 and Ser473 [103].

It is worth noting that the relationship between miRs and PTEN signaling can determine tumor stage and its advancement. In GC cells in which miR-205-5p undergoes upregulation, while a reduction occurs in expression of PTEN, tumor cells are in advanced stages. Downregulation of miR-250-5p enhances expression of PTEN, which mediates apoptosis induction and tumor-growth inhibition [104]. Taking everything into account, studies demonstrate that oncogene miRs bind to 3′-UTR of PTEN to negatively affect its expression. The decrease in expression of PTEN leads to the resistance of GC cells into apoptosis, their enhanced proliferation, high metastatic capability, and poor prognosis of patients with GC (Table 1) [99,105].

#### 5.1.2. MicroRNAs and PTEN Inhibition

To date, just two studies have identified an onco-suppressor miR that affects PTEN signaling by exerting its antitumor activity, so we include it here. Phosphatidylinositol 3,4,5- trisphosphate RAC exchanger 2a (P-Rex2a) is a guanine nucleotide exchange factor (GEF) and interacts with PTEN to induce PI3K in cancer cells [115]. MiR-338-3p affects P-Rex2a in suppressing GC progression. MiR-338-3p binds to P-Rex2a and reduces its expression. This impact triggers PTEN signaling that, in turn, suppresses PI3K/Akt, leading to a decrease in proliferation and invasion of GC cells [116]. MiR/PTEN axis can activate pro-apoptotic factors in GC therapy. MiR-370 enhances expression of PTEN to inhibit Pi3K/Akt/mTOR signaling, resulting in the upregulation of caspase-3, p53, and GSK-3μ and subsequent induction of apoptosis in GC cells [117].

### 5.2. MicroRNAs, PTEN, Drug-Therapy, and Radio-Therapy

Although much attention has been directed toward the adverse effects of chemotherapy, there is another emerging problem in cancer therapy, known as “drug resistance” [118,119]. How to overcome drug resistance is under discussion, and different methods have been mentioned, such as using poly-chemotherapy, genetic intervention, finding novel chemotherapeutic agents, and so on [120,121,122]. It is quite obvious that the best way to accelerate the pace for the aforementioned strategies is to reveal the molecular pathways involved in drug resistance. In this section, we provide a new insight into the involvement of miRs in regulating PTEN in the drug resistance of GC cells.

Cisplatin resistance is a troublesome problem for chemotherapy [123,124]. It is worth noting that factors that contribute to cisplatin resistance have been identified in recent years by extensive researches [125,126]. In GC cells, the miR/PTEN axis is of importance. Normally, cisplatin administration in patients with GC should improve their overall survival by induction of apoptosis in GC cells and reducing their survival and viability. However, it is held that miR-21, as an oncogene factor in GC cells, is an impediment against cisplatin-mediated apoptosis. This is due to the downregulation of PTEN by miR-21 and subsequent resistance of GC cells into cisplatin-induced apoptosis [127]. Therefore, decreasing the expression of such miRs can pave the road to the inhibition of drug resistance in GC cells. In a recent experiment, it was found that silencing miR-147 results in enhanced sensitivity of GC cells into 5-fluorouracil chemotherapy via PTEN upregulation. In fact, by overexpression of PTEN, a decrease occurs in PI3K/Akt that subsequently suppresses proliferation and triggers apoptosis in GC cells [128]. The involvement of the miR/PTEN axis in the drug resistance of GC cells can be attributed to their malignant behavior. To be more specific, when more cancer cells are at an advanced stage, the more they are resistant into chemotherapy [129,130]. Such a phenomenon can be associated with the miR/PTEN axis. In GC cells, miR-21-5p decreases PTEN expression. As it was mentioned in the introduction section, partial loss of PTEN leads to tumorigenesis and directing tumor masses into advanced stages. In this case, downregulation of PTEN by miR-21-5p triggers doxorubicin resistance via promoting malignant behavior of GC cells and facilitating their progression into advanced stages [131]. Taking everything into account, it appears that oncogene miRs such as miR-193-3p, -19a/b, -21, and -106a contribute to resistance of GC cells to chemotherapy via downregulation of PTEN and promoting their proliferation by activation of the PI3K/Akt signaling pathway [132,133,134,135].

In addition to chemoresistance, cancer cells are able to obtain resistance to radiotherapy. This feature of cancer cells has challenged the efficacy of combination therapy with chemotherapy and radiotherapy [136,137]. Thus, finding molecular pathways involved in the radio-resistance of GC cells is of importance in another dimension. Similarly, miR/PTEN participates in the radio-resistance of GC cells. It has been reported that overexpression of miR-221 and miR-222 occurs in GC cells. These miRs are able to bind into 3′-UTR of PTEN to inhibit its expression. Consequently, a decrease occurs in the expression of PTEN and provides the condition for the uncontrolled proliferation and migration of GC cells. This leads to the resistance of GC cells to radiotherapy [138]. Further studies can focus on downstream targets of the miR/PTEN axis in radiotherapy of GC cells.

### 5.3. CircularRNAs Target PTEN

CircularRNAs (circRNAs) are another kind of RNA molecule with a different structure from linear RNAs. In contrast to linear RNAs that have a 5′ cap and 3′ tail, circRNAs are closed-loop structures with covalent bonds that have 5′-3′ polarities or polyadenylated tails [139]. After the discovery of circRNAs in 1970, it was believed that these kinds of RNAs emanate from errors in alternative splicing, since they have low expression [140,141,142]. However, further research demonstrated that circRNAs are a distinct type of RNA molecule with modulatory effects on cellular events [143,144,145,146]. In the case of PTEN signaling, different experiments have shown that circRNAs are able to act as upstream mediators of the PTEN signaling pathway in cancer cells [147,148,149]. This section is devoted to investigating the role of circRNAs in the regulation of PTEN in GC cells.

In GC cells, circRNAs can regulate PTEN to dually inhibit or promote the proliferation and invasion of cancer cells. A newly published experiment has shown that circRNA ZFR is an oncogene factor that reduces expression of miR-101-3p to enhance the malignancy of cancer cells [150]. A similar phenomenon occurs in GC cells, so that circRNA ZFR regulates miR expression to affect PTEN signaling. By binding into miR-130a and miR-107, circRNA ZFR reduces their expression to facilitate overexpression of PTEN in GC cells. Then, a decrease occurs in the viability of GC cells via upregulation of PTEN and stimulation of apoptosis [151]. On the other hand, there are circRNAs that downregulate PTEN signaling in ensuring malignant behavior of GC cells. CiRS-7 is considered to be an oncogene circRNA in GC cells that considerably elevates growth by sponging miR-7 and subsequent activation of HOXB13 [152]. Moreover, CiRS-7 can promote the migration of cancer cells by downregulation of miR-1299 and upregulation of matrix metalloproteinases (MMPs) [153]. These studies demonstrate the stimulatory effect of CiRS-7 on cancer cells, and a same role is observed in GC cells via targeting the PTEN/PI3K/Akt signaling pathway. In GC cells, CiRS-7 downregulates the expression of miR-7 as an onco-suppressor factor. Therefore, the PI3K/Akt signaling pathway is activated via PTEN downregulation, leading to the increased proliferation and invasion of GC cells [154].

CircRNA LARP4 is another onco-suppressor factor in cancer. This circRNA suppresses both growth and metastasis of cancer cells via downregulation of miR-424-5p [155]. In respect to antitumor role of LARP4, low expression of this circRNA is correlated with poor prognosis of patients with cancer [156]. Therefore, enhancing expression of circRNA LARP4 can provide condition for a decrease in viability of cancer cells [157]. There is a reverse relationship between circRNA LARP4 and miR-1323 in GC cells. MiR-1323 expression demonstrates a decrease by activity of LARP4. Then, PTEN signaling is activated to suppress the PI3K/Akt axis, resulting in the decreased progression of GC cells [158]. MiR-130a-3p is another oncogene factor in GC cells, and its expression is affected by onco-suppressor circGRAMD1B. This circRNA reduces expression of miR-130a-3p via sponging to promote expression of PTEN. Then, the condition for interaction of PTEN and p21 is provided to suppress the malignancy of GC cells [159]. Overall, these studies are in agreement with the fact that circRNAs are efficient upstream mediators of PTEN signaling in GC cells, and further studies should be performed to reveal more circRNAs capable of regulating PTEN.

### 5.4. LncRNAs Target PTEN

In addition to miRs and circRNAs, the role of lncRNAs in the regulation of PTEN in GC cells has been investigated. LncRNAs are ncRNAs with more than 200 nucleotides and biological functions in both normal and cancerous cells [160,161,162]. LncRNAs-mediated regulation of PTEN is of importance in cancer cells, and understanding the relationship between lncRNAs, PTEN signaling, and mediators can broaden our insight toward their regulation in further experiments, and suppressing cancer malignancy [66,163,164]. In this section, our aim is to provide a mechanistic discussion about regulation of PTEN by lncRNAs in GC cells. In the previous section, we demonstrated that circRNAs and miRs are able to function as upstream mediators of PTEN in GC cells. The role of lncRNAs in regulation of PTEN in GC cells has been more examined compared to circRNAs. Thus, it would be possible to discuss more molecular pathways.

The expression of onco-suppressor lncRNAs undergoes downregulation in cancer cells. LncRNA SLC25A5-AS1 has low expression in GC cells, and this leads to a decrease in apoptotic cell death. SLC25A5-AS1 reduces the expression of miR-19a-3p via acting as an endogenous competitor. MiR-19a-3p decreases the response of GC cells to apoptosis via downregulation of PTEN and subsequent activation of the PI3K/Akt signaling pathway. SLC25A5-AS1 induces PTEN expression via miR-19a-3p downregulation, to interfere with activation of the PI3K/Akt signaling pathway. This directs GC cells toward apoptosis [146]. In fact, onco-suppressor lncRNAs protect PTEN from repression by negatively targeting miRs. For instance, lncRNA PTENP1 functions as competing endogenous RNAs (ceRNAs) to downregulate miR-106b and miR-93. As a consequence, PTEN is activated to exert its inhibitory effect of GC cells [165].

LncRNAs can lead to enhanced metastasis of GC cells by inhibition of PTEN. This can be related to downstream targets of PI3K/Akt that play a significant role in enhancing the migratory ability of cancer cells. As we mentioned earlier, the migratory ability of cancer cells is significantly accelerated by the EMT mechanism [166]. Slug is one of the regulators of EMT, and its upregulation can stimulate EMT and provide invasion of cancer cells [167]. LncRNA GPR65-1 is an oncogene factor in GC cells that can stimulate the migration of GC cells. LncRNA GPR65-1 stimulates Akt signaling via PTEN downregulation. Then, slug undergoes upregulation in GC cells to induce invasion and metastasis of GC cells [168]. These studies demonstrate that (1) lncRNAs are efficient upstream mediators of PTEN in GC cells, (2) they can affect upstream mediators of PTEN such as miRs, and (3) they dually affect proliferation and invasion of GC cells. Table 2 summarizes lncRNAs regulating PTEN in GC cells.

## 6. Antitumor Compounds Target PTEN

In the previous sections, we provided a comprehensive discussion about regulation of PTEN signaling by epigenetic factors such as miRs, circRNAs, and lncRNAs. Among them, lncRNAs and miRs have been studied more than circRNAs, showing that there is still a long way to go for finding the exact relationship between circRNAs and PTEN in GC cells. However, it seems that epigenetic factors are not the only ones that can regulate PTEN signaling in GC cells. Increasing evidence highlights that fact that antitumor compounds, particularly natural products, are capable of regulation of PTEN in different cancers [177,178]. This story has been repeated for GC, and the aim of this section is to shed some light on regulation of PTEN in GC cells by antitumor compounds.

Curcumin is a polyphenol and natural product isolated from root of turmeric rhizome [179,180,181]. It has pharmacological activities for the amelioration of cardiovascular disorders [182], improving blood lipids [183], reducing the risk of diabetes development [184,185], and so on. However, among them, antitumor activity of curcumin is of importance, since curcumin has been able to suppress proliferation of cancer cells, induce apoptosis, and inhibit their migration [167,186,187]. Curcumin can negatively affect proliferation of GC cells via targeting PTEN signaling. In affecting PTEN signaling, curcumin can target its upstream mediator, miR-21. Inhibition of GC proliferation by curcumin is time- and dose-dependent. Administration of curcumin (5–40 μM) reduces expression of miR-21 to activate PTEN signaling (highest effect in high doses of curcumin). Then, Akt undergoes downregulation to suppress proliferation of GC cells [188]. To date, just one study has evaluated the efficacy of curcumin in targeting PTEN signaling in GC cells, and more studies will reveal different molecular pathways of curcumin action in targeting PTEN in GC cells.

Resveratrol (Res) is also a polyphenol agent and well-known in traditional Chinese medicine [189,190]. It is extracted from *Polygonum cuspidatum* and exclusively applied as a chemotherapeutic agent in the treatment of different cancers [191]. The antitumor activity of Res emanates from its effect on different molecular pathways, such as Wnt [192], miRs [193,194], and so on. Two studies have investigated the antitumor activity of Res against GC via targeting PTEN signaling. Administration of Res (100–400 μM) results in a decrease in p-PTEN (inactive) form in a dose-dependent manner (highest effect at 200 μM). This leads to suppression of the PI3K/Akt signaling pathway and subsequent stimulation of cell cycle arrest at G0/G1 phase [195]. As it was mentioned earlier, EMT and the migratory ability of cancer cells have a positive relationship [196,197]. EMT activation is correlated with the resistance of cancer cells to chemotherapy [198,199]. Thus, the inhibition of EMT is of interest in sensitizing cancer cells into inhibitory effects of chemotherapeutic agents. Administration of Res (50 and 100 mg/L) induces PTEN to downregulate PI3K/Akt signaling. Then, epithelial marker E-cadherin level demonstrates an increase, while levels of vimentin and β-catenin as mesenchymal markers decrease, showing the inhibition of EMT by Res as a result of PTEN activation. This leads to enhanced sensitivity of GC cells to doxorubicin chemotherapy (Table 3) [200].

In regard to the regulation of PTEN by antitumor agents in GC therapy, the following outcomes may occur:

- The antitumor compounds are able to affect PTEN in a time- and dose-dependent manners,
- They can induce PTEN signaling [201,202,203],
- They suppress the PI3K/Akt signaling pathway [204],
- They are capable of regulating upstream mediators of PTEN, such as HDAC1 [205],
- They inhibit the development of chemoresistance in GC cells [206],
- And, finally, they interfere with proliferation and invasion of GC cells via targeting PTEN [207].

## 7. Upstream Modulators of PTEN

### 7.1. Proliferation of Gastric Cancer Cells

Cytotoxin-associated gene A (CagA) is considered to be a factor related to gastric disorders such as peptic ulcer disease [213]. Clinical studies have also confirmed the involvement of CagA in gastrointestinal disorders, so that, in an experiment conducted by Weel and colleagues, it was found that 85–100% of patients with duodenal ulcer have an infection with CagA+ *Helicobacter pylori* (*H. pylori*) [214]. The role of CagA in GC has been associated with its effect on PTEN. The expression of PTEN undergoes downregulation in GC cells and tissues, while CagA has high expression in these malignant cells. Investigation of molecular signaling pathways demonstrates that CagA reduces expression of PTEN by enhancing DNA methylation and directing GC cells into uncontrolled proliferation [215]. Arginine/serine-rich coiled coil 1 (RSRC1) gene has been located on chromosome 3, and it encodes a protein containing arginine and serine components. The products of this gene are involved in cellular evens and are able to modulate transcription process [216,217]. RSRC1 participates in suppressing the uncontrolled growth of GC cells via upregulation of PTEN [218]. Thus, enhancing expression of RSRC1 as an upstream mediator of PTEN can be considered as a potential strategy in GC therapy. In suppressing the proliferation of GC cells, PI3K/Akt, as a downstream target of PTEN, should be inhibited. Downregulation of PTEN in GC cells occurs as a result of its phosphorylation at S380/T382/T383 cluster. Upstream mediators of PTEN signaling with onco-suppressor activity such as PDZK1 are able to inhibit PTEN phosphorylation, enhance its stability, and suppress the PI3K/Akt signaling pathway, resulting in the reduced proliferation of GC cells [219].

In addition to having physiological roles such as embryogenesis and T-cell activation, WWP2′s oncogene role in cancer cells is being revealed by accumulating data. It is an E3 ligase of PTEN and contributes to carcinogenesis via PI3K/Akt activation [220,221,222,223,224,225,226,227,228]. In GC cells, WWP2 enhances the growth and viability of cancer cells via the downregulation of PTEN. Silencing of WWP2 leads to stimulation of PTEN and subsequent a decrease in the proliferation of GC cells [229]. It is worth mentioning that most of the studies have focused on onco-suppressor mediators of PTEN in GC. Krüppel-like factors (KLFs) are able to affect different cellular events via acting as transcription regulators [230,231,232]. The exact role of KLFs in cancers has not been understood, and it seems that KLFs function as oncogene or onco-suppressor factors in respect to the tissue context, the cancer type, or its stage [233]. However, KLFs exert antitumor activity via the regulation of PTEN. The in vitro and in vivo experiments demonstrate that KLFs impair the PI3K/Akt signaling pathway via PTEN upregulation, to induce apoptosis and cell cycle arrest in GC cells [234]. Taking everything into account, studies exhibit that upstream mediators of PTEN are able to disrupt viability and growth of GC cells, and more studies are needed to clarify the relationship between PTEN and its upstream mediator, as well as to find more upstream mediators [235,236,237,238].

### 7.2. Metastasis of Gastric Cancer Cells

Note that there have been studies focusing on the relationship between PTEN and its upstream mediators in metastasis of GC cells. In fact, the process of metastasis accounts for a significant number of deaths among different cancer patients, including those suffering from gastric cancer [239,240]. Each experiment has evaluated a novel axis that is of importance for effective GC therapy. In this section, we provide an integrated discussion about the regulation of PTEN by these molecular pathways.

Ten-Eleven Translocation 1 (TET1) is an enzyme contributing to the conversion of 5-methylcytosine to 5-hydroxymethylcytosine. TET1 participates in demethylation of CpG islands and, in this way, activates its downstream targets. Increasing evidence demonstrates the loss of TET1 in different cancers, particularly GC [241,242,243,244,245]. Overexpression of TET1 inhibits Akt signaling via PTEN downregulation to suppress metastasis of GC cells [246]. The metastasis of GC cells is associated with poor survival of patients. Suppressing an invasion of GC cells not only improves overall survival of patients with GC, but can also promote the efficacy of antitumor agents in eradicating cancer cells. Therefore, there is an urgent need for revealing molecular pathways involved in metastasis of GC cells and their further targeting. As it was mentioned earlier, EMT is associated with the migration of cancer cells [44,93]. In GC cells, the interaction between EZH2 and PTEN signaling provides the condition for enhancing invasion. EZH2 attaches to the promoter of PTEN to decrease its expression. Then, the EMT mechanism is activated via Akt signaling [247]. The EZH2/PTEN/Akt/EMT axis can be targeted in further studies to overcome GC.

As a potential factor in cell fate, increasing evidence has shown that Notch signaling plays like a double-edged sword in cancer, and it has both oncogene and onco-suppressor activities [248,249,250,251,252,253,254]. It has been reported that metastasis of GC cells is tightly regulated by Notch/PTEN/Akt axis. In promoting the invasion of GC cells, Notch signaling stimulates Akt via PTEN downregulation. Using siRNA, Notch signaling was inhibited, leading to a dramatic reduction in metastasis of GC cells [255]. Studies related to PTEN regulation reveal that oncogene factors downregulate PTEN, to induce the PI3K/Akt signaling pathway. A further step is related to the downstream targeting of Akt signaling, so that, as it was mentioned in this section, EMT can be induced to ensure metastasis of cancer cells. More studies will provide more underlying molecular pathways in this process [124,256].

### 7.3. Drug Resistance of Gastric Cancer Cells

The regulation of PTEN by upstream molecular pathways can also lead to the emergence of chemoresistance of malignant gastric cancer cells. Akt is also known as PKB and can support cancer cells against apoptotic cell death. Increasing evidence demonstrates that Akt-induced pro-survival pathways mediate chemoresistance of cancer cells [257,258,259]. The overexpression of Akt is correlated with the downregulation of PTEN. This provides a condition for resistance of GC cells to chemotherapeutic agents such as 5-fluorouracil, Adriamycin, mitomycin, and cis-platinum [260]. To date, just one study has evaluated the regulation of PTEN by other molecular pathways (except miRs, lncRNAs, and circRNAs) in the induction of chemoresistance. More studies are required to discover novel pathways involved in the chemoresistance of GC cells via PTEN regulation.

## 8. Activation and Deactivation of PTEN in Gastric Cancer

**Figure 2:**
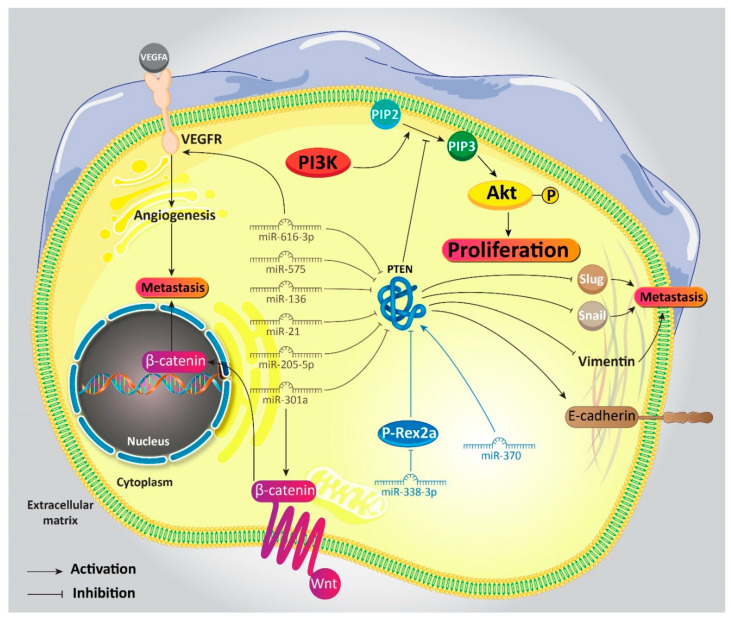
Activation/inactivation of PTEN in gastric cancer and its involvement in proliferation and metastasis of these malignant cells.

With respect to the role of PTEN in suppressing both proliferation and invasion of GC cells, any impairment in this signaling can lead to tumorigenesis. We mentioned in the introduction that a partial loss in PTEN expression can result in the development of different cancers. This story is also true for GC. In previous sections, we mechanistically investigated the regulation of PTEN in GC cancer. It is quite obvious that PTEN downregulation enhances the proliferation of GC cells, and one of the most common downstream targets of PTEN is the PI3K/Akt signaling pathway. It has been reported that inactivation of PTEN in GC cells leads to the stimulation of PI3K/Akt signaling and subsequent increase in proliferation [261]. The PTEN loss is a positive factor for enhanced invasion of GC cells [262]. Activation of PTEN signaling remarkably diminishes tumor growth [263]. Moreover, enhancing the expression of PTEN can pave the road to suppressing the migration of GC cells [264]. Clinical studies have confirmed the role of PTEN in the detection of GC. It seems that PTEN loss ensures the advanced stage of GC [265]. All of these studies demonstrate that (1) PTEN is an onco-suppressor factor, and (2) modulation of its expression is of importance in GC therapy (Figure 2) [266,267].

## 9. Conclusions and Remarks

What we discussed in this work is the onco-suppressor role of PTEN signaling. PI3K/Akt is the most common downstream target of PTEN in suppressing both proliferation and invasion of cancer cells. However, other molecular pathways such as FAK can be targeted by tumor suppressor PTEN. MiRs, lncRNAs, and circRNAs are able to function as upstream mediators of PTEN in GC cells. Moreover, antitumor compounds can affect PTEN (upregulation) to inhibit proliferation and invasion of GC cells. The interesting point is the relationship between PTEN and EMT mechanism in GC cells. As it was mentioned, migration of GC cells into neighboring and distant cells and tissues significantly reduces overall survival of patients with cancer. PTEN can inhibit the EMT mechanism by targeting PI3K/Akt signaling pathway that we described in the text. It seems that we are at the beginning point of knowing the molecular pathways by which PTEN affects proliferation and malignancy of GC cells. Furthermore, there is still much space for progress about understanding new upstream mediators of PTEN signaling. SiRNA and CRISPR/Cas9, as powerful editing tools, can be used to modulate the expression of PTEN in GC cells. Newer antitumor drugs can also be introduced into the regulation of PTEN in GC cells.

## Figures and Tables

**Table 1:** Regulation of PTEN and its downstream targets by miRs in gastric cancer (GC) cells.

MiR	Molecular Signaling	Results	References
MiR-28	PTEN/PI3K/Akt	Downregulation of PTEN Stimulation of PI3K/Akt Promoting invasion and proliferation	[106]
MiR-21	PTEN/Akt	Inhibition of PTEN Stimulation of Akt Enhancing Bcl-2/Bax ratio	[107,108]
MiR-26a MiR-106b MiR-214	PTEN	Decreasing PTEN expression Poor prognosis Low survival	[109,110,111]
MiR-21	PTEN/TGF-β/EMT	Downregulation of PTEN Induction of TGF-1/EMT axis Enhancing E-cadherin levels Reducing N-cadherin and vimentin levels Increasing migration of cancer cells	[112]
MiR-214	PTEN	Downregulation of PTEN Reducing cell cycle arrest	[113]
MiR-301a	PTEN/Wnt/β-catenin	Suppressing PTEN Activation of Wnt signaling Promoting metastasis	[114]

**Table 2:** LncRNAs regulating PTEN in GC cells and their relationship with proliferation and invasion.

LncRNA	Signaling Network	Results	References
PCAT18	MiR-107/PTEN/PI3K/Akt	Downregulation of miR-107 by PCAT18 Stimulation of PTEN signaling Inhibition of PI3K/Akt Inducing tumor growth inhibition	[169]
TUBA4B	MiR-214/PTEN MiR-216a/b-PTEN	Downregulation of miR-214 and miR-216a/b Stimulation of PTEN Suppressing proliferation and migration	[170]
DGCR5	MiR-23b/PTEN	Inhibition of miR-23b Stimulation of PTEN Induction of apoptosis	[171]
LINC00470	PTEN	Reducing PTEN stability and directing it into degradation Enhancing proliferation and invasion	[172]
AFAP1-AS1	PTEN/Akt	Reducing PTEN expression Stimulation of Akt Downregulation of pro-apoptotic factors PARP1, capase-3 and caspase-9	[173]
HOTAIRM1	MiR-17-5p/PTEN	Inhibition of miR-17-5p expression via sponging Upregulation of PTEN Decreasing viability and proliferation	[174]
FER1L4	MiR-106a-5p/PTEN	Inhibition of miR-106a-5p Stimulation of PTEN Disrupting proliferation	[175]
HOTAIR	MiR-17-5p/PTEN	Upregulation of miR-17-5p by HOTAIR Inhibition of PTEN Enhancing chemoresistance	[167]
PCAT1	EZH2/PTEN	Increasing EZH2 expression Downregulation of PTEN Stimulation of cisplatin resistance	[176]

**Table 3:** Antitumor compounds targeting PTEN in GC therapy.

Antitumor Agent	Concentration and Time	In Vitro/In Vivo	Results	References
Geridonin	10 μM for 24 h	In vitro (MGC 803 cell line)	Stimulation of PTEN Downregulation of PI3K/Akt signaling Accumulation of p53 Stimulation of apoptosis	[208]
Ursolic acid	0–35 μM for 24 h	In vitro (SGC-7901 cells)	PTEN activation Translocation of cofilin-1 from cytoplasm to mitochondria Stimulation of apoptosis	[209]
Baicalein	0–80 μM for 24 and 48 h	In vitro (AGS cells)	Activation of PTEN Suppressing hypoxia mediated Akt Inhibition of glycolysis	[210]
Genistein	10–80 μmoL/L for 24 h	In vitro (SGC-7901 and BGC-823 cells)	Upregulation of PTEN Stimulation of G2/M arrest	[211]
Methylxanthine	Different doses (in vitro) for 12 and 24 h 4 and 8 mmoL/L for 24 days	In vitro (MGC-803 cells) In vivo (model of GC)	Stimulation of PTEN Inhibition of PI3K/Akt/mTOR signaling Suppressing proliferation and migration	[212]

## Abbreviations

PTEN	phosphatase and tensin homolog
GC	gastric cancer
PIP2	phosphatidylinositol 4-5-diphosphate
TRPV4	transient receptor potential vanilloid type 4
NF-B	nuclear factor-kappaB
lncRNA	long non-coding RNA
pCR	pathologic complete response
ncRNAs	non-coding RNAs
EMT	epithelial-to-mesenchymal transition
GEF	guanine nucleotide exchange factor
circRNAs	circular RNAs
MMPs	matrix metalloproteinases
ceRMAs	competing endogenous RNAs
Res	resveratrol
CagA	cytotoxin-associated gene A
*H. pylori*	*Helicobacter pylori*
RSRC1	arginine/serine-rich coiled coil-1
KLFs	Krüppel-like factors
TET1	Ten-Eleven Translocation 1
